# Accuracy of Internet-Based Patient Self-Report of Postdischarge Health Care Utilization and Complications Following Orthopedic Procedures: Observational Cohort Study

**DOI:** 10.2196/10405

**Published:** 2018-07-20

**Authors:** Benjamin I Rosner, Marc Gottlieb, William N Anderson

**Affiliations:** ^1^ HealthLoop Inc Mountain View, CA United States; ^2^ Department of Hospital Medicine Kaiser Permanente Santa Clara, CA United States; ^3^ Anthem Inc Atlanta, GA United States

**Keywords:** patient-generated health data, patient reported outcome measures, patient self-report, complications, utilization, patient readmission, emergency room, hospital economics

## Abstract

**Background:**

The accuracy of patient self-report of health care utilization and complications has yet to be determined. If patients are accurate and engaged self-reporters, collecting this information in a manner that is temporally proximate to the health care utilization events themselves may prove valuable to health care organizations undertaking quality improvement initiatives for which such data are often unavailable.

**Objective:**

The objective of this study was to measure the accuracy of patient self-report of health care utilization and complications in the 90 days following orthopedic procedures using an automated digital patient engagement platform.

**Methods:**

We conducted a multicenter real-world observational cohort study across 10 orthopedic practices in California and Nevada. A total of 371 Anthem members with claims data meeting inclusion criteria who had undergone orthopedic procedures between March 1, 2015, and July 1, 2016, at participating practices already routinely using an automated digital patient engagement platform for asynchronous remote guidance and telemonitoring were sent surveys through the platform (in addition to the other materials being provided to them through the platform) regarding 90-day postencounter health care utilization and complications. Their self-reports to structured survey questions of health care utilization and complications were compared to claims data as a reference.

**Results:**

The mean age of the 371 survey recipients was 56.5 (SD 15.7) years, 48.8% (181/371) of whom were female; 285 individuals who responded to 1 or more survey questions had a mean age of 56.9 (SD 15.4) years and a 49.5% (141/285) female distribution. There were no significant differences in demographics or event prevalence rates between responders and nonresponders. With an overall survey completion rate of 76.8% (285/371), patients were found to have accuracy of self-report characterized by a kappa of 0.80 and agreement of 0.99 and a kappa of 1.00 and agreement of 1.00 for 90-day hospital admissions and pulmonary embolism, respectively. Accuracy of self-report of 90-day emergency room/urgent care visits and of surgical site infection were characterized by a kappa of 0.45 and agreement of 0.96 and a kappa of 0.53 and agreement of 0.97, respectively. Accuracy for other complications such as deep vein thrombosis, hemorrhage, severe constipation, and fracture/dislocation was lower, influenced by low event prevalence rates within our sample.

**Conclusions:**

In this multicenter observational cohort study using an automated internet-based digital patient engagement platform, we found that patients were most accurate self-reporters of 90-day hospital admissions and pulmonary embolism, followed by 90-day surgical site infection and emergency room/urgent care visits. They were less accurate for deep vein thrombosis and least accurate for hemorrhage, severe constipation, and fracture/dislocation. A total of 76.8% (285/371) of patients completed surveys without the need for clinical staff to collect responses, suggesting the acceptability to patients of internet-based survey dissemination from and collection by clinical teams. While our methods enabled detection of events outside of index institutions, assessment of accuracy of self-report for presence and absence of events and nonresponse bias analysis, low event prevalence rates, particularly for several of the complications, limit the conclusions that may be drawn for some of the findings. Nevertheless, this investigation suggests the potential that engaging patients in self-report through such survey modalities may offer for the timely and accurate measurement of matters germane to health care organizations engaged in quality improvement efforts post discharge.

## Introduction

Rates of health care utilization and complications post discharge are topics of increasing interest and value under both federal [1-3] and commercial [4,5] bundled payment and hospital readmission reduction initiatives [6-8]. However, accurate and timely measurement and reporting of these outcomes vary, in part due to limitations of the sources from which such data are derived, large variations in the ways in which they are measured [9], and the lag time between the capture of these events in reporting systems and their dissemination back to the very health care organizations at which the index encounters occurred [10]. In the rise of the era of the patient-as-partner-in-care, health care organizations engaged in quality improvement or those seeking to enhance performance in value-based reimbursement models may find patients to be uniquely valuable sources of information regarding rates of health care utilization and complications post discharge. However, for a feedback cycle between patients and health care organizations to be meaningful and useful in quality improvement, health care organizations need to (1) enable and engender patient participation in this cycle, (2) scale the low-burden dissemination of such surveys to patients, (3) attain high survey completion rates, and (4) feel confident that patients can be timely and accurate self-reporters.

This study aimed to understand whether patients in real-world clinical practice settings, surveyed at 90 days post encounter through an automated digital patient engagement (DPE) platform, are accurate self-reporters of real-time or near real-time readmissions, emergency room/urgent care (ERUC) usage, and postencounter complications.

Studies have been conducted investigating the accuracy of patient self-report on topics ranging from past medical history [11,12], surgical history [13], and diagnosis underlying the need for a given intervention [14]. Investigations have also described the accuracy of self-reported complications using either general practitioner surveys [15] or independent surgeon review of confirmatory studies [16] or medical records [17] as references. Some of these studies have been confounded by the methodological tautology of relying on patients to confirm their own self-reports [16,17], and others have been limited in their completeness by assessing accuracy only among patients reporting the presence of events without also assessing accuracy of those reporting the absence of events [15-17]—the latter being a cohort that is much larger when examining low prevalence events such as readmissions and complications, and arguably just as important from a quality improvement perspective.

Other studies have been prone to the potential for recall error, sometimes referred to as memory decay, caused by the lag time between when the event occurred and the relatively distant time at which the patient was later surveyed for such events [17,18]. Stability of accurate patient recall for such events appears to remain over at least 2 to 3 months [19,20] but suffers from notable decline between 3 and 8 months [21,22], suggesting that earlier survey intervals may be beneficial for capturing accurate response data.

Determining the accuracy of patient self-report is confounded by several additional factors. First, index institutions are only implicitly aware through their own reporting systems of readmissions back to their own health care systems. It has been reported, for example, that leakage—presentation of the patient to facilities other than the index facility for complications and readmissions—occurs in 31% to 65% of cases with some rates as high as 87.5%, suggesting that index institutions have large blind spots about postencounter health care utilization for which they may bear financial risk [16,17,23]. This degree of leakage, in a health care environment such as that of the United States which lacks a single payer, means that readmissions, complications, and health care utilization may be underrecognized and underreported. Although large public payers such as Medicare may be able to report readmission rates back to index facilities with reasonable leakage-free accuracy, Medicare beneficiaries are not demographically representative of the US population at large and constitute a portion of the US population that is increasingly being outpaced in certain procedural volume areas by other age cohorts [24]. Second, reliance solely on metrics such as proportion of correct reports [15], concordance [16,17,25], or agreement [13] that inflate when event prevalence rates are low rather than presenting these metrics alongside of an appropriate kappa statistic may lead to misinterpretations of the accuracy of patient self-report.

Some studies have attempted to address leakage by using single [17] or multi-institutional [25] databases or registries. Notable among them is Harrold et al [18], who used medical records from the Function and Outcomes Research for Comparative Effectiveness in Total Joint Replacement (FORCE-TJR) network, a group of over 230 surgeons across 28 states, to evaluate the accuracy of patient self-report following total knee and total hip replacement against data from hospitals within the region of the index facility as the reference. The study also evaluated the accuracy of patients reporting no utilization by examining orthopedic notes at the FORCE-TJR core sites as well as emergency department, day surgery, and hospitalization records at the index site. For 60% of the patients, the nearest hospitals to their homes were not the index facilities, so the investigators received releases of information from the nearest hospitals to the patients’ homes in 87% of cases. Nevertheless, utilization or complications documented in primary care or other specialty settings or in external facilities not otherwise included may have posed limitations.

Two parties, the payers and the patient, may be the most knowledgeable about health care utilization and complication events and in ideal positions to report the most comprehensive health care event information post encounter. If quality improvement is a goal, and readmissions and postencounter complications represent the last mile in the health care quality chain, could patients become active participants by providing accurate and timely information about these outcomes back to health care organizations? While payer databases may be nearly leakage-free references against which to compare patient self-report, relatively few have been used in such analyses, and when they have, they have been largely limited to single-payer settings [26-28] or to employer-based health care claims data [29] that do not necessarily generalize to the broader population.

To our knowledge, this is the first study of the accuracy of patient self-report using a commercial payer claims database as a reference that (1) minimizes the potential for underreporting due to leakage across specialties, care settings, and institutions, (2) enables measurement of self-report among patients attesting to either the presence or absence of events, (3) includes a nonresponse bias analysis, and (4) facilitates the timely collection of responses to mitigate recall error using workflow-compatible tools such as automated internet-based DPE platforms to survey patients in an optimal postencounter timeframe.

## Methods

### Study Design

We conducted a multicenter observational cohort study on postdischarge outcomes following orthopedic procedures falling into 1 of 5 categories: hip arthroplasty, knee arthroplasty, knee arthroscopic procedures, shoulder arthroscopic procedures, and knee arthrotomy. As part of a broader investigation of the impact of automated DPE platforms on health care costs and outcomes [30], data on patient self-report of 90-day hospital admissions, ERUC use, and complications were collected through an automated DPE platform (HealthLoop Inc) and compared against claims data from Anthem Inc for Anthem members who had undergone the procedure at 1 of 10 community orthopedic practices in California and Nevada between March 1, 2015, and July 1, 2016. These community practices ranged in size from solo practitioner to multispecialty practices with as many as 25 physicians.

We followed the Reporting of Studies Conducted Using Observational Routinely-Collected Data (RECORD) statement checklist (an extension of the Enhancing the Quality and Transparency of Health Research [EQUATOR] Network Strengthening the Reporting of Observational Studies in Epidemiology [STROBE] guidelines) [31,32] and the Standards for Quality Improvement and Reporting Excellence (SQUIRE) guidelines [33]. Although this was not a randomized controlled trial, we adhered to as many of the Consolidated Standards of Reporting Trials (CONSORT)-EHEALTH checklist items (v1.6.1) as appropriate [34,35]. This study received a determination of exemption from human subjects research by E&I Review Services, an independent institutional review board.

### Enrollment

Practices whose patients were sent utilization and complication surveys were already using the DPE platform in their routine provision of care to provide asynchronous, automated remote guidance and conduct telemonitoring before and after procedures. Because this was a retrospective observational cohort study and not a prospective trial, there was no recruitment. Patients were enrolled for routine clinical purposes on the platform that was already in use at practice sites (ie, they were not enrolled in a trial), and investigators later compared deidentified survey responses to claims data. Enrollment of patients on the platform was at the discretion of the individual practices and was not within the influence of the authors. However, since practices were using the platform for their routine provision of care, most patients undergoing relevant procedures at these sites were enrolled on the platform and were receiving surveys. The only exclusion criteria at the points of care were the lack of a valid email address and internet access, as required to receive check-in notifications and interact with the DPE platform itself.

### Digital Patient Engagement Platform and Health Care Utilization and Complication Surveys

For context, the DPE platform worked as follows. Automated email check-in notifications generated by the platform and designed to come from the physicians were sent to patients longitudinally over time according to predetermined procedure-specific care plan schedules. A notification link within the email prompt took the patient into the Health Insurance Portability and Accountability Act (HIPAA)-compliant environment where materials pertinent to that day, written to a Flesch-Kincaid 6th grade reading level, were queued up, including reminders, checklists, educational materials, structured symptom assessments, and patient reported outcome measure (PROM) surveys. At approximately 90 days post encounter, the utilization and complication questions pertinent to this study were asked of patients (see Multimedia Appendix 1).

With regard to health care utilization, patients were asked about hospital admissions and ERUC visits. They were also queried about complications relevant to their procedures, including deep vein thrombosis (DVT), pulmonary embolism (PE), surgical site infection (SSI, including sepsis), hemorrhage (including gastrointestinal bleeding), fracture/dislocation, and severe constipation. Delivery of surveys and collection of responses were fully automated, requiring no additional human support. Further DPE platform details have been described in Steele [36] and Rosner [30]. The platform was accessible to patients and health care professionals via the internet on desktop, laptop, tablet, and iOS- or Android-enabled mobile devices.

### Data Inclusion and Exclusion

Claims outcomes were identified in the Anthem database with *International Classification of Diseases, Ninth Revision, Clinical Modification* (ICD-9-CM) and ICD-10-CM codes. Because the transition from ICD-9 to ICD-10 occurred during the study period, forward and reverse mappings between the versions were performed. All data extracted from the Anthem database were deidentified with randomized case identifiers applied for investigator reference.

**Figure 1:**
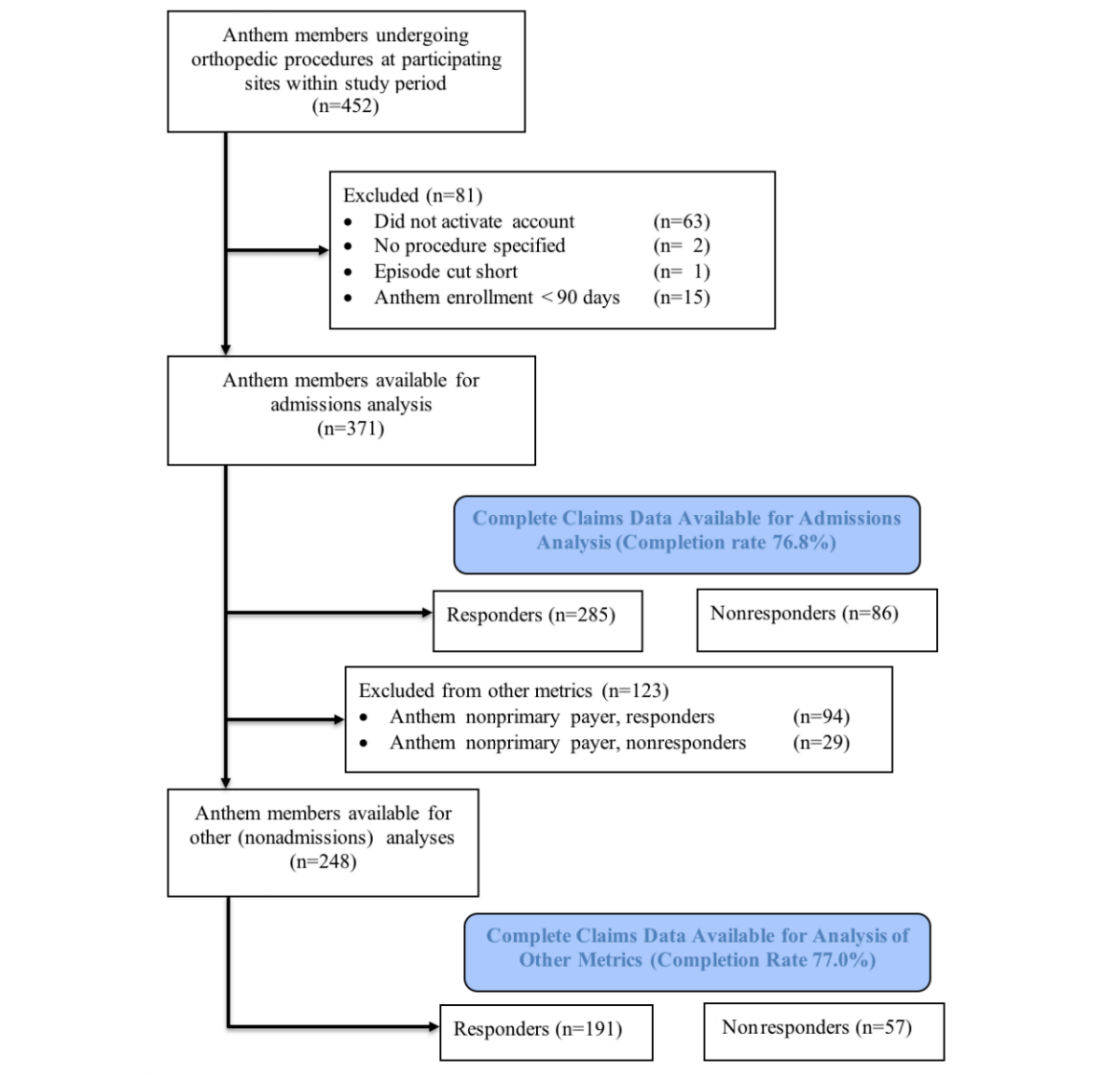
Data inclusion and exclusion waterfall.

Not considered for the study were practice patients who were not Anthem members, because we did not have claims data against which to compare patient responses. Among the Anthem members receiving surveys, excluded from analysis were patients not having a procedure within 1 of the 5 specified categories, patients whose enrollment with Anthem terminated prior to 90 days (ie, incomplete 90-day claims data), and patients with short episode durations, defined as patients who had more than 1 eligible procedure within a 90-day timeframe whose 90-day surveys for the first procedure could have overlapped with and been confounded by events associated with the second procedure (Figure 1). Response data from patients with capitated health maintenance organization products for which Anthem did not have full professional service claims were also to be excluded, but after the above exclusions, there were no remaining patients for whom this applied.

For admissions outcomes, the Anthem database contained complete data even for patients for whom Anthem was not the primary payer. However, for other outcomes (eg, ERUC visits and complications), the Anthem database did not necessarily contain full data for patients whose primary payer was not Anthem. Therefore, for outcomes other than admissions, data from patients for whom Anthem was not the primary payer were further excluded from analysis.

### Primary Analysis

**Figure 2:**
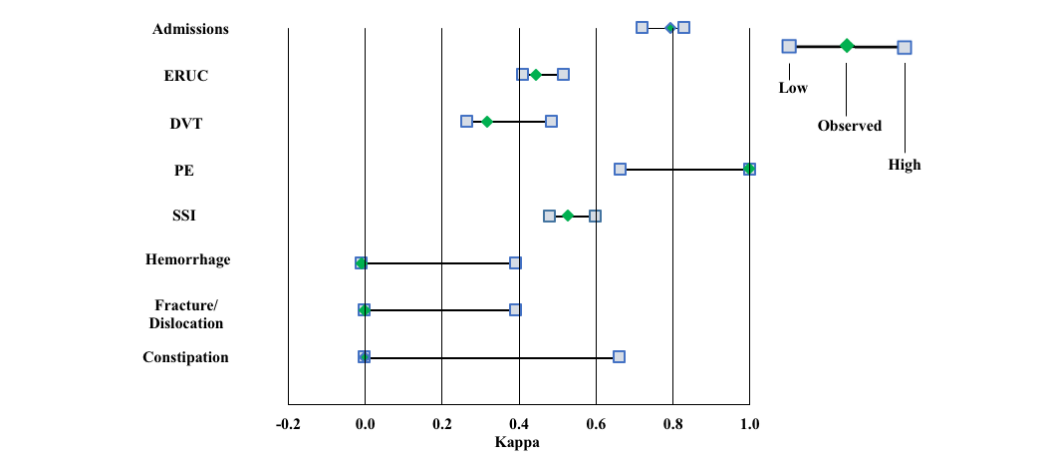
Sensitivity of observed kappa to changing 1 true negative to false positive and to changing 1 true negative to true positive. DVT: deep vein thrombosis; ERUC: emergency room/urgent care; PE: pulmonary embolism; SSI: surgical site infection.

The primary outcome metric for each survey question was the kappa statistic, a standard measure of how much the observed agreement between patient self-report and the events in the claims database differed from expected. Other metrics of relevance, widely reported in related studies, included true positive (TP), true negative (TN), false positive (FP), false negative (FN), sensitivity, specificity, positive predictive value, negative predictive value, and agreement, the latter of which was defined as (TP+TN)/(TP+TN+FP+FN). Although some studies use agreement as the primary outcome metric to evaluate the accuracy of patient self-report, agreement is prone to inflation when event prevalence rates are low, and kappa serves as a standard statistic that is not unduly influenced in this manner. Many studies have used the following thresholds as guidance to help interpret the meaning of the value of kappa, but there is not universal agreement as to what cut points should be considered clinically meaningful, and as such, interpretation of kappa in a relative sense is more useful than in an absolute sense: kappa <0.20, poor; 0.20 to 0.39, fair; 0.40 to 0.59, moderate; 0.60 to 0.79, very good; and ≥0.80, excellent [37-39]. A sensitivity analysis of the primary outcome (Figure 2) was also performed to illustrate the influence on kappa of changing 1 TN response to TP and 1 TN response to FP for each question.

#### Secondary Analyses

We conducted 3 secondary analyses to examine for potential bias that could influence kappa, the primary outcome metric. When it was not possible to evaluate for bias influencing kappa (eg, when there were no self-report data from which to calculate kappas from cohorts such as account nonactivators or survey nonresponders), we considered differences in surrogate metrics such as event prevalence rates or demographics. The 3 demographic variables to which the authors had access were age; gender; and DxCG score, a composite indicator of overall illness burden.

In the first among these, a nonactivator bias analysis, we examined for bias between patients who activated their platform accounts and those who did not. Since kappas were not available for comparison (nonactivators, by definition, did not furnish self-report data from which kappas could be calculated), we examined for differences in demographics between these cohorts. We did not examine for differences in event prevalence rates in this bias analysis since it has been shown in the literature [30] that one of the effects of DPE platforms for patients who activate their accounts is to reduce event rates relative to those who do not.

We similarly conducted a nonresponse bias analysis, examining for differences in demographics between activated patients who responded to self-report surveys and those who did not. Again, differences in kappa could not be examined because the nonresponders furnished no self-report data, but evaluation for differences in event prevalence rates was possible because account activation status in both cohorts was the same.

Finally, we conducted a bias analysis between 2 cohorts with expected demographic differences: the arthroplasty cohort (expected to be older) and the nonarthroplasty cohort. Since age and comorbidity burden are known to drive event rates, differences in event rates would not necessarily be an accurate assessment of bias. However, in this analysis, since both cohorts did furnish self-report data, we were able to directly assess for differences in kappa as a function of demographics.

#### Statistical Analysis

Analyses were performed in R version 3.2.3 (The R Foundation). Fisher exact test and analysis of variance were used where categorical and continuous variables were compared, respectively. *P*<.05 was deemed significant. Kappas were computed using the CohenKappa function from the R DescTools package and compared using the values and standard errors produced by that function. The kappa statistic was considered significant if the confidence interval excluded 0 [40].

## Results

### User Statistics

The mean age of the 371 survey recipients available for admission analysis was 56.5 (SD 15.7) years, 48.8% (181/371) of whom were female. The mean DxCG score for this group was 5.32 (SD 5.28). The mean age of the 285 Anthem members who responded to the surveys was 56.9 (SD 15.4) years, 49.5% (141/285) of whom were female (Table 1). The mean DxCG score for the 285 responders was 4.89 (SD 4.96). As a measure of overall platform usage (not just survey response rates) within the responder cohort, the mean patient engagement, measured as the number of check-ins performed divided by the number of check-ins scheduled, with additional credit in both the numerator and denominator for proactive, unscheduled activity in the platform, was 79.7% (SD 19.9). There was no statistical difference in overall platform usage as measured by engagement between patients less than 65 years of age and those 65 years and older (*P*=.61).

### Primary Analysis

Surveys were sent to 452 Anthem members, of whom 371 met the inclusion criteria (Figure 1). Of these 371 patients, 285 completed 1 or more survey questions and submitted the surveys regarding admissions (76.8% completion rate [41]). Regarding all other survey questions (for which availability of complete claims data required patients to have Anthem as a primary payer), 123 patients for whom Anthem was not the primary payer were excluded from analysis. Of these patients, 65.0% (80/123) had Medicare as their primary payer. Among the patients for whom Anthem was the primary payer, 248 met the inclusion criteria, and 191 completed 1 or more questions and submitted the survey (77.0% completion rate).

With regard to 90-day admissions, patient self-reports were found to be characterized by a kappa of 0.80 and agreement of 0.99 (Table 2). With respect to ERUC, patient responses were found to be characterized by a kappa of 0.45 and agreement of 0.96. Regarding complications, patient responses were characterized by kappas and agreements of 1.00 and 1.00 for PE, 0.53 and 0.97 for SSI, 0.32 and 0.97 for DVT, 0.00 and 0.98 for fracture/dislocation, 0.00 and 0.99 for severe constipation, and –0.01 and 0.98 for hemorrhage, respectively.

### Secondary Analyses

In the nonactivator bias analysis, we found there were no significant demographic differences between the patients who activated their DPE platform accounts and those who did not. The mean ages in the activated versus nonactivated cohorts were 56.2 (SD 15.7) and 55.8 (SD 18.5) years, respectively (*P*=.88). The gender distributions in the activated versus nonactivated cohorts were 48.4% (188/388) female and 45.3% (29/64) female, respectively (*P*=.69). The mean DxCG scores in the activated versus nonactivated cohorts were 5.30 (SD 5.25) and 5.18 (SD 5.89), respectively (*P*=.87).

In the nonresponse bias analysis, there were no significant demographic differences between patients who responded and those who did not. The mean ages in the responder versus nonresponder cohorts were 56.5 (SD 15.4) years and 55.3 (SD 17.5) years, respectively (*P*=.46). The gender distributions in the responders versus nonresponders were 49.5% (149/301) female and 45.0% (68/151) female, respectively (*P*=.42). The mean DxCG scores in the responders versus nonresponders were 4.96 (SD 5.27) and 5.91 (SD 5.44), respectively (*P*=.08). The lowest *P* value for event prevalence rate differences between the 2 groups was .30 (Table 3).

**Table 1:** Demographic characteristics of responders.

Characteristic		Value
**90-day admission (all payers, n=285)**		
	Age (years), mean (SD)	56.9 (15.4)
	Age (years), median (IQR^a^)	58 (47, 68)
	Female, n (%)	141 (49.5)
	DxCG^b^, mean (SD)	4.89 (4.96)
	DxCG, median (IQR)	3.58 (2.02, 6.25)
**90-day events (Anthem primary payer, n=191)**		
	Age (years), mean (SD)	49.0 (12.3)
	Age (years), median (IQR)	52 (42, 58)
	Female, n (%)	92 (48.2)
	DxCG, mean (SD)	3.99 (4.95)
	DxCG, median (IQR)	2.87 (1.59, 4.77)

^a^IQR: interquartile range.

^b^DxCG: a composite indicator of overall illness burden.

**Table 2:** Counts and calculated values for determination of accuracy of patient self-report.

Characteristics		TP^a^	FP^b^	TN^c^	FN^d^	Prevalence	Sn^e^	Sp^f^	PPV^g^	NPV^h^	Agreement	Kappa (95% CI)
**90-day admission (all payers)**												
	Admission	4	2	239	0	0.02	1.00	0.99	0.67	1.00	0.99	0.80 (0.52 to 1.00)^i^
**90-day events (Anthem primary)**												
	Emergency room/urgent care visit	3	7	148	0	0.02	1.00	0.95	0.30	1.00	0.96	0.45 (0.11 to 0.78)^i^
	Pulmonary embolism	1	0	120	0	0.01	1.00	1.00	1.00	1.00	1.00	1.00 (1.00 to 1.00)^i^
	Surgical site infection	3	3	149	2	0.03	0.6	0.98	0.50	0.99	0.97	0.53 (0.17 to 0.89)^i^
	Deep vein thrombosis	1	1	115	3	0.03	0.25	0.99	0.50	0.97	0.97	0.32 (–0.17 to 0.81)
	Fracture/dislocation	0	3	152	0	0.00	N/A^j^	0.98	0.00	1.00	0.98	0.00 (0.00 to 0.00)
	Severe constipation	0	1	154	0	0.00	N/A	0.99	0.00	1.00	0.99	0.00 (0.00 to 0.00)
	Hemorrhage	0	2	154	1	0.01	0.00	0.99	0.00	0.99	0.98	–0.01 (–0.02 to 0.00)

^a^TP: true positive.

^b^FP: false positive.

^c^TN: true negative.

^d^FN: false negative.

^e^Sn: sensitivity.

^f^Sp: specificity.

^g^PPV: positive predictive value.

^h^NPV: negative predictive value.

^i^Indicates statistical significance.

^j^N/A: not applicable.

**Table 3:** Nonresponder bias analysis.

Characteristics		Responder (n)	Responder prevalence	Nonresponder (n)	Nonresponder prevalence	*P* value
**90-day admissions (all payers)**						
	Admissions	245	0.02	40	0.00	>.99
**90-day events (Anthem primary)**						
	Emergency room/urgent care visit	158	0.02	33	0.00	>.99
	Pulmonary embolism	121	0.01	70	0.00	>.99
	Surgical site infection	157	0.03	34	0.03	>.99
	Deep vein thrombosis	120	0.03	71	0.00	.30
	Fracture/dislocation	155	0.00	36	0.00	>.99
	Severe constipation	155	0.00	36	0.00	>.99
	Hemorrhage	157	0.01	34	0.00	>.99

In comparing arthroplasty to nonarthroplasty cohorts, we found no difference in gender distribution between the 2 groups (female 51.4% [53/103] vs 48.4% [88/182], respectively, *P*=.62). As expected, the arthroplasty patients were older (mean age of 66.7 (SD 10.2) years versus 51.3 (SD 15.0) years, respectively, *P*<.001) and had higher mean DxCG scores (7.36 [SD 5.70] versus 3.49 [SD 3.85], respectively, *P*<.001). However, despite the age and DxCG differences, there were no significant differences between these groups in kappa for any of the questions (lowest *P* value .09), suggesting that differences in age and illness burden across these cohorts did not have effects on self-report accuracy.

## Discussion

### Principal Findings

In this multicenter observational cohort study, we sought to assess the accuracy of patient self-report of health care utilization and complications in the 90 days post encounter following 5 types of orthopedic procedures. We found the accuracy of patient self-report of 90-day hospital admissions and 90-day ERUC visits to be characterized by kappas of 0.80 and 0.45, respectively. These findings are consistent with those of Ungar [42] (kappas of 0.80 and 0.60, respectively), who described parental report of pediatric asthma-related hospitalizations and emergency room visits in a Canadian population, and Yu [28] (kappas of 0.75 and 0.52, respectively), who described self-report of utilization of such services among a general Taiwanese population.

We also found the accuracy of patient self-report of 90-day PE events, SSI, DVT, fracture/dislocation, severe constipation, and hemorrhage to be characterized by kappas of 1.00, 0.53, 0.32, 0.00, 0.00, and –0.01, respectively, although the interpretation of several of these items may be limited in our study by small sample sizes for events with extremely low prevalence rates. For example, the limitation related to fracture/dislocation is demonstrated, when sample size was larger in a New Zealand registry study [43], by the close agreement observed between patient self-report of hip dislocation and revision in the 6 months following hip arthroplasty and registry confirmed hip dislocation and revision (0.37% vs. 0.39%, respectively). This limitation is further demonstrated by a sensitivity analysis (Figure 2) in which we examine the kappa when 1 TN is changed to TP and when 1 TN is changed to FP. The kappa is shown to be particularly sensitive to events of lowest prevalence in which there are either no TPs or FPs in our sample (severe constipation, fracture/dislocation, hemorrhage, and PE). The implication, particularly for the first 3, is that due to low event prevalence, our results may not have sufficient resolution to conclude that patients are necessarily poor self-reporters of these specific complications.

It is noteworthy that accuracy appears quite high for some items and lower for others, even when event prevalence is not negligible. One explanation that has been suggested is the concordance between patient self-report and a reference is higher for significant events such as hospitalizations than for more routine events [29]. Our results may be consistent with that explanation. Nevertheless, it is noteworthy that 2 patients had false positive reports of admissions. One explanation is that while these patients may have been accurate in reporting an overnight stay in the hospital, for billing purposes they might have been classified as outpatients under observation status or under the Centers for Medicare and Medicaid Services’ 2-midnight rule. To minimize the impact that clinical decision units or observation units might have on false positives, our survey question for hospital admissions (Multimedia Appendix 1) asked patients not to count overnight stays in the emergency department. Not all clinical decision units or observation units are physically located in emergency departments, however, and it may be difficult for patients to know or even later ascertain whether their stay in such a unit or their stay in the hospital for less than 2 midnights had been classified by the payer as inpatient or outpatient.

Regarding the lower accuracy of self-report observed for ERUC than for hospital admissions, the difference between payer classification and patient perception of what constitutes an urgent care visit may be central. It has been reported, for example, that patients may consider an urgent care visit to be either to an urgent care center or a general practitioner for a same-day visit. On the other hand, payer claims data differentiate the services based on location and would attribute the general practitioner visit as an outpatient encounter rather than an urgent care encounter [29]. Therefore, it is arguable that these 2 visit types should not be aggregated within a single survey question and that location should be more distinctly specified. This hypothesis seems to be supported by the high agreement and high kappas reported by Harrold et al [18] regarding emergency room visits post discharge in which the emergency room was the only location specified in the survey question.

Accuracy of self-report may also suffer due to survey question language around concepts that are well understood to medical practitioners but not to others. For example, Greenbaum [17] found that there was good concordance for clearly defined complications (eg, pulmonary embolism, dislocation) and poor concordance for less clearly defined complications (eg, major bleeding). Similarly, Bream [9] reported that accuracy of SSI self-reporting was variable, but there was greater accuracy when patients were asked about symptoms or antibiotic use (as we have done) rather than being asked about an overall diagnosis. This suggests that the limitations may not reside with the patient’s actual capacity for accuracy but with the language and construct of the questions. Such language should be within the grasp and availability of the lay person, although for certain medical concepts, this may not be possible. When the language is put into lay terminology and in the context of phenomena within the patient experience, accuracy may be optimized.

Also of relevance are the intervals at which patients are asked to self-report. Although short intervals (eg, 30 days) might be desirable from a recall perspective, cumulative event rates at 30 days are likely to be low and less useful to health care organizations than event rates accumulated over longer periods of time. Furthermore, administering surveys at high frequencies and comparing them to references at recurring intervals such as 30, 60, and 90 days may be prohibitively resource intensive using traditional means. As such, many studies have asked patients for self-report at a single 6-month time point [16,17,44]. However, such a long lag between a health care utilization or complication event and the survey itself may introduce recall error. In several studies, stability of patient recall appears to remain over 2 to 3 months [19,20] but suffers from marked decline between 3 and 8 months [21,22]. Survey periods of 90 days, as in this study, may not only minimize recall error but facilitate accuracy and timeliness of results in closed loop feedback cycles to index institutions engaged in quality improvement.

Accuracy of patient self-report is just 1 component critical to postencounter quality improvement processes for health care organizations. Beyond accuracy are needs for easy distribution and collection of surveys, timely reporting to ensure a temporally proximate feedback cycle, and high rates of patient response. In this study, we report a 76.8% completion rate of 90-day surveys facilitated entirely through an automated process, requiring no additional manual support. Automated DPE platforms may offer a practical and scalable distribution and collection modality acceptable to patients and health care teams.

### Strengths

This study has overcome several limitations of prior, related studies in that it (1) mitigated the potential for underreporting due to leakage across specialties, care settings, and institutions, (2) enabled the measurement of self-report among patients attesting to the presence and absence of events—the latter being a major challenge in studies involving manual chart review because the cohort of patients without events is substantially larger than those with events—and (3) facilitated the timely collection of responses to mitigate recall error using workflow compatible tools.

Using a payer claims database overcame limitations inherent in a commonly applied technique of using physician retrospective report as a reference, an approach that has been described as prone to underreporting due to poor professional compliance with completing audit data, inaccurate coding of procedures [44], unawareness due to leakage [18,25], and potential for bias [45].

Another strength was the use of 90-day self-report surveys rather than longer periods commonly used such as 6 months [16,17,44], as it has been demonstrated that accuracy of self-report begins to taper after 2 to 3 months [20,21]. Finally, unlike many studies which fail to conduct nonresponse bias analyses—including those that acknowledge the potential of nonresponse bias in their own samples [15]—we did conduct such an analysis and found there to be no nonresponse bias in our sample.

### Limitations

Accuracy of claims data is subject to the accuracy of coding, which is reportedly variable [46,47]. Low prevalence rates of some events in our sample also limit resolution of the results for several of the survey questions. The influence of demographic factors such as age, race, ethnicity, socioeconomic status, and level of education on the use of internet and email and on the potential for inaccuracy of self-report is worthwhile to consider. Several studies have demonstrated differences in the use of email and internet according to race and ethnicity [48] and based on age, with the most notable age-based use drop-off among those over 75 years [48,49]. The only demographic factors to which the authors had access, however, were age, gender, and DxCG score. Subgroup analysis based on age alone was not possible because the low prevalence rates of events in our sample combined with exclusion of data for patients whose primary payer was not Anthem (particularly those 75+ years of age for whom internet and email usage is reported to drop) rendered most subgroup analyses too small to lead to any meaningful conclusions. However, we did explore the influence of age and disease burden by comparing the older arthroplasty group to the younger nonarthroplasty group and found no difference in accuracy of self-report between these groups. An additional limitation was that comprehensibility of survey questions around concepts that are inherently clinical may be a factor in this sort of investigation. Although we made every attempt to put questions in terms within patients’ grasp, some concepts will likely always be challenging for patients to self-report, either because the definition of an event is clinical or the patient does not have access to all of the information needed to self-report (eg, lab values, imaging studies, classification of a 1-night hospital stay as either inpatient or outpatient).

Generalizability is often a key issue in translating study findings to real-world practice. Our findings came from a limited set of orthopedic patients in a West Coast US geographic area and may not necessarily generalize to other patient populations, geographies, and medical conditions. However, there are other aspects of our study that may contribute toward generalizability. First, rather than being limited to a single site, this was a multicenter study that drew from community orthopedic practices. Second, while the lack of real-world practice results has often been criticized among digital health applications [50], this retrospective study occurred in real-world practice settings and did not involve recruitment, formal inclusion or exclusion criteria, or research staff to support or promote patient engagement.

### Conclusions

We have demonstrated through the use of an internet-based automated DPE platform in real-world clinical settings kappa and agreement values for patient self-report of 90-day hospital admission of 0.80 and 0.99, respectively, and of 90-day ERUC visits of 0.45 and 0.96, respectively. We have also demonstrated higher accuracy for major complications such as PE and lower accuracy for complications such as hemorrhage, which were found to be subject to low event prevalence rates and small sample sizes. We further demonstrated a survey completion rate of 76.8%, requiring no additional support in real-world clinical practice settings, and that there was no significant bias introduced by platform nonactivators, survey nonresponders, or patients of older age or higher disease burden.

These findings may bear relevance to the very health care entities that are increasingly bearing risk under programs such as the Hospital Readmissions Reduction Program and bundled payment programs including the Comprehensive Care for Joint Replacement program, the Bundled Payments for Care Improvement initiative [1], and its recent successor, Bundled Payments for Care Improvement Advanced [2]. These institutions have had limited temporally proximate insight into readmission and postdischarge complication rates for their own patients, in part because of leakage and in part because of the lag between when an event happens and when reconciliation occurs. Patient self-report of utilization and complications has been considered not only as a means of enhancing the accuracy and timeliness of utilization and complication reporting but as a potential means of engaging the patient further as a partner in his or her own care.

As health care facilities consider such self-report mechanisms as means to enhance their own quality improvement efforts, capture of health care events by index institutions is only part of the needed solution. It remains up to these institutions to implement quality improvement initiatives that reduce potentially avoidable readmissions and complications based on the closed feedback cycle. Additional research spanning other medical specialties, geographies, and patient populations may demonstrate whether this approach could be generalized more broadly.

## Appendix

Multimedia Appendix 1 Utilization and complications questionnaire.

## Abbreviations

CONSORT: Consolidated Standards of Reporting Trials
DPE: digital patient engagement
DVT: deep vein thrombosis
DxCG: a composite indicator of overall illness burden
EQUATOR: Enhancing the Quality and Transparency of Health Research
ERUC: emergency room/urgent care
FN: false negative
FORCE-TJR: Function and Outcomes Research for Comparative Effectiveness in Total Joint Replacement
FP: false positive
HIPAA: Health Insurance Portability and Accountability Act
IQR: interquartile range
ICD-9-CM: International Classification of Diseases, Ninth Revision, Clinical Modification
PE: pulmonary embolism
PROM: patient reported outcome measure
RECORD: Reporting of Studies Conducted Using Observational Routinely-Collected Data
SQUIRE: Standards for Quality Improvement and Reporting Excellence
SSI: surgical site infection
STROBE: Strengthening the Reporting of Observational Studies in Epidemiology
TN: true negative
TP: true positive
