# Supplementary material for: Accuracy of Internet-Based Patient Self-Report of Postdischarge Health Care Utilization and Complications Following Orthopedic Procedures: Observational Cohort Study
Source: J Med Internet Res. 2018 Jul 20;20(7):e10405. doi: 10.2196/10405 (PMC6076369; doi:10.2196/10405)
Supplement: Multimedia Appendix 1 [file jmir_v20i7e10405_app1.pdf]

Utilization and Complications Questionnaire

The following survey questions were sent to patients approximately 90-days post-encounter. In addition to the pure Yes or No answer choices, there were some qualified answers choices, such as "Yes, but I'm not sure if the infection was related to my procedure" as pertained to surgical site infection. In order to compare to the claims database, it was appropriate and straightforward to treat most qualified Yes or No answers shown below as Yes or No, respectively. However, the above response choice for surgical site infection, as well as all response choices of "I'm not sure," were treated as "unknown" and thereby missing for analysis.

1. Hospital admission
   Since your procedure, did you have an unplanned hospital admission (an overnight stay in the hospital, not counting an overnight stay in the emergency room)?
   - No, I did not
   - Yes, 1 time
   - Yes, 2 times
   - Yes, 3 times
   - Yes, 4 or more times

2. Emergency room/urgent care visits
   Since your procedure, did you have any unexpected visits to an emergency room or an urgent care clinic?
   - No, I did not
   - Yes, 1 time
   - Yes, 2 times
   - Yes, 3 times
   - Yes, 4 or more times

3. Surgical site infection
   Since your procedure, were you prescribed an antibiotic by a doctor for an infection that was related to your procedure?
   - No, I was not
   - No, but I did take an antibiotic as prescribed to prevent one
   - Yes, but I'm not sure if the infection was related to my procedure
   - Yes, I was

4. Deep vein thrombosis
   Since your procedure, did a doctor diagnose you as having a new blood clot in the arm or leg (also known as a deep vein thrombosis, or DVT)?
   - No
   - I'm not sure
   - Yes

5. Hemorrhage
   Since your procedure, did you need to receive treatment from a doctor for bleeding that was unexpected and that you were not able to control on your own?
   - No - & I wasn’t taking a prescription blood thinner at the time
   - No - & I was taking a prescription blood thinner at the time
   - Yes - & I wasn’t taking a prescription blood thinner at the time
   - Yes - & I was taking a prescription blood thinner at the time

6. Pulmonary embolism
   Since your procedure, did a doctor diagnose you as having a new blood clot in the lungs (also known as a pulmonary embolism, or PE)?
   - No
   - I’m not sure
   - Yes

7. Fracture/dislocation
   Since your procedure, did a doctor confirm that you had fractured a bone or dislocated a joint that was on the same arm or leg as where you had your procedure?
   - No
   - I’m not sure
   - Yes

8. Severe constipation
   Since your procedure, did you have to see a doctor for treatment for severe constipation related to prescription pain medication use?
   - No
   - I’m not sure
   - Yes
